# Restoration of HCV-Specific Immune Responses with Antiviral Therapy: A Case for DAA Treatment in Acute HCV Infection

**DOI:** 10.3390/cells8040317

**Published:** 2019-04-05

**Authors:** Julia L. Casey, Jordan J. Feld, Sonya A. MacParland

**Affiliations:** 1 Institute of Medical Science, University of Toronto, Toronto, ON M5S 1A8, Canada; julia.casey@mail.utoronto.ca (J.L.C.); Jordan.Feld@uhn.ca (J.J.F.); 2 Toronto General Hospital Research Institute, University Health Network, Toronto, ON M5G 2C4, Canada; 3 Departments of Laboratory Medicine & Pathobiology and Immunology, University of Toronto, Toronto, ON M5S 1A1, Canada

**Keywords:** Hepatitis C Virus, direct acting antivirals, DAAs, interferon, IFN, immune restoration, immune exhaustion, exhausted T cells

## Abstract

Worldwide, 71 million individuals are chronically infected with Hepatitis C Virus (HCV). Chronic HCV infection can lead to potentially fatal outcomes including liver cirrhosis and hepatocellular carcinoma. HCV-specific immune responses play a major role in viral control and may explain why approximately 20% of infections are spontaneously cleared before the establishment of chronicity. Chronic infection, associated with prolonged antigen exposure, leads to immune exhaustion of HCV-specific T cells. These exhausted T cells are unable to control the viral infection. Before the introduction of direct acting antivirals (DAAs), interferon (IFN)-based therapies demonstrated successful clearance of viral infection in approximately 50% of treated patients. New effective and well-tolerated DAAs lead to a sustained virological response (SVR) in more than 95% of patients regardless of viral genotype. Researchers have investigated whether treatment, and the subsequent elimination of HCV antigen, can reverse this HCV-induced exhausted phenotype. Here we review literature exploring the restoration of HCV-specific immune responses following antiviral therapy, both IFN and DAA-based regimens. IFN treatment during acute HCV infection results in greater immune restoration than IFN treatment of chronically infected patients. Immune restoration data following DAA treatment in chronically HCV infected patients shows varied results but suggests that DAA treatment may lead to partial restoration that could be improved with earlier administration. Future research should investigate immune restoration following DAA therapies administered during acute HCV infection.

## 1. Introduction

### 1.1. Natural History of Hepatitis C Virus (HCV) Infection

The World Health Organization (WHO) estimates the prevalence of chronic hepatitis C virus (HCV) infection to be 71 million people worldwide [1]. Annually, 39,000 deaths are attributed to HCV infection [1]. These deaths are predominantly caused by outcomes of chronic infection including cirrhosis and hepatocellular carcinoma [1]. Approximately 15%–45% of those infected with HCV spontaneously clear viremia with the remaining 55%–85% advancing to chronic Hepatitis C (CHC) [2]. HCV chronicity is generally defined as HCV RNA positivity beyond 6 months [3], but is truly marked by the exhaustion of the immune response leading to failure of spontaneous HCV clearance [2]. Chronic HCV infection is usually a lifelong infection unless treated. Currently there is no preventative or therapeutic vaccine and treatment targets patients who are in the chronic phase of HCV infection and generally not those recently infected with HCV [4]. 

### 1.2. Virus-Induced Immune Dysfunction

The outcome of a viral infection can be either acute infection followed by control of viral replication and spontaneous clearance or viral persistence resulting in a chronic infection. Both host and viral characteristics influence the acute versus chronic outcome [5,6]. Many different cell types contribute to the elimination of viral infections such as HCV [7]. Both innate and adaptive immune responses are active during an HCV infection; however, in most cases HCV manages to escape these responses to establish chronicity. 

The high variability in disease outcomes between immune competent individuals is not well understood, but several factors have been identified that influence an individual’s ability to control HCV viral infection. Female sex, Aboriginal ethnicity [8] and younger age [9] are associated with increased rates of HCV clearance. In contrast, human immunodeficiency virus (HIV) coinfection and injection drug use are associated with increased HCV persistence [8]. Breadth, but not frequency, of HCV-specific CD8^+^ T cell responses are shown to be reduced in HIV/HCV co-infected individuals [10]. However, with improved treatment of both HIV and HCV infections, sustained virological response (SVR) outcomes for coinfected individuals demonstrate similar success [11]. Furthermore, a retrospective investigation of HCV infection found spontaneous clearance of HCV was more frequently documented among subjects with a history of icteric hepatitis and hepatitis B virus (HBV) coinfections [12]. 

Variation in genes involved in the host immune response have been found to influence an individual’s ability to clear acute HCV infection. The rs12979860 CC genotype, a single nucleotide polymorphism (SNP) upstream of the *IL28B* gene, which encodes the type III interferon IFN-λ has been shown to be a strong predictor of an effective response to IFN therapy for chronic HCV infection [5]. Individuals with the rs12979860 CC genotype are more likely to spontaneously clear HCV infection and respond to pegylated-interferon (PEG-IFN)-α/ribavirin(RBV) treatment [5,13,14]. The association between rs12979860 CC genotype and spontaneous resolution of HCV infection has been shown among individuals of both European and African ancestry [5] and confirmed in a Chinese population [15]. The major C allele of rs12979860 SNP in the general population has a frequency of 0.23–0.55 among Africans, 0.53–0.80 among Europeans and 0.66–1.00 among Asians [16]. Accordingly, Asians and Europeans demonstrate higher response rates to combined peg-IFN-α and ribavirin therapy than African-descendants [17]. The strong correlation between this genotype, ethnic groups and control of HCV suggests the presence of a selective pressure exerted by environmental factors [18]. 

Following an inability to spontaneously clear HCV infection, the majority of cases succumb to a chronic infection [19]. Natural killer (NK) cells and macrophages play an important role in innate immune responses and become impaired in chronic HCV infection [20]. During chronic infection, activity of NK cells has also been reported to be impaired in patients with HCV infection [20]. The ability of NK cells to produce and secrete IFN-γ is weakened after exposure to HCV-infected cells [21]. The decline of IFN-γ production is consistent with the reduction of NK cell degranulation, demonstrating a reduced functional capacity of these NK cells [21]. The inhibition of NK cell function was associated with downregulation of NK-activating receptors on NK cell surfaces [21]. Inhibition of ex vivo NK functions with HCV infected cells corresponds with reduced surface expression of the natural cytotoxicity receptor NKp30 [22]. HCV NS5A protein has been shown to induce IL-10 and TGF-β secretion in monocytes [23]. These cytokines also have a regulatory, suppressive effect on NK cell function. 

HCV-specific CD8^+^ T cells or cytotoxic T lymphocytes (CTLs) are required for the control of HCV infection [24]. The CTL response to acute HCV infection begins with the effector phase when naïve T cells are primed by HCV antigen, undergo expansion and facilitate clearance of infection by specifically killing infected cells. In the case of spontaneous clearance, this response is successful at clearing the HCV infection [25]. A small subset of these HCV-specific CD8^+^ T cells will persist as long-lived memory cells that can be recalled upon re-exposure to the same pathogen [25]. Although the development of an effector CTL response should result in clearance of HCV, in most instances HCV manages to escape immune control and becomes persistent [25]. If antigen stimulation remains high, the result can be exhaustion, characterized by an inability to develop and mount a functional CTL response [25]. However, the mechanism contributing to HCV-specific CD8^+^ T-cell failure and the resulting persistence of HCV infection is still poorly understood. Complex molecular pathways, including transcriptional differences, direct the differentiation of CD8^+^ T cells into memory or exhausted T cells during HCV infection [26]. Memory T cells during spontaneously resolved HCV infection share co-regulated T cell identity genes while exhausted T cells share different co-regulated T cell identity genes, distinguishing the two populations [26]. The cytokines secreted by T cells during viral replication influence the final outcome of HCV infection. The role of CD4^+^ have also been explored during HCV infection suggesting the presence of HCV-specific CD4^+^ T cell responses to be common in spontaneously resolved HCV infection [27,28,29,30]. A more recent study found the majority of peripheral HCV-specific CD4^+^ T cells in patients with self-limiting HCV infection had an effector phenotype (CD4^+^CD25^high^CD134^+^CD39^−^) with high IFN-γ production, while the HCV-specific CD4^+^ T cells from patients who progressed to a chronic HCV infection were dominated by a regulatory phenotype (CD4^+^CD25^high^CD134^+^CD39^+^) and high IL-10 production [31]. It has also been shown that the majority of liver-infiltrating T cells in chronic HCV infection are Type 1 Helper T (T_h_1) cells able to secrete IFN-γ, but unable to secrete IL-4 or IL-5 [32]. 

As seen in other chronic viral infections [33,34,35,36,37], persistent infection with HCV results in T cell exhaustion [38]. HCV-related exhaustion leads to weak or absent HCV-specific antiviral T cell responses. T cell exhaustion develops in a step-wise and progressive manner, varies in severity, and results in blunted virus-specific immunity [39]. These include an impaired ability to respond to viral peptide and to mitogen. Exhausted T cells have impaired proliferation abilities and reduced secretion of antiviral cytokines including IL-2 [40], IFN-γ [37,41], IL-21 [42] and TNF-α [41,43]. These T cells also express high levels of exhaustion markers, PD-1 [44,45,46,47], TIM-3 [48,49,50,51,52] and CTLA-4 [53,54] and low levels of memory markers such as interleukin 7 receptor (CD127) [46,55,56,57] and Bcl-2 [58]. Co-expression of PD-1 with 2B4, CD160 and KLRG1 has been shown on exhausted T cells during HCV infection [59]. Additionally, murine models have demonstrated signaling through TIGIT is critical for maintaining chronic exhaustion during prolonged viral infection [60]. The presence of TIGIT has been shown on exhausted T cells during viral infection and its expression positively correlates with disease progression [61]. In chronic HCV infection, this exhausted immune phenotype is present and presumed to be the reason for failure to clear viral infection [62]. In this review, we provide background on HCV immune dysfunction and review literature exploring the restoration of HCV-specific immune responses with antiviral therapy, both IFN-based and more recent DAA therapies. 

### 1.3. Treatment of HCV Infection

The main goal of HCV therapy is to achieve a sustained virological response (SVR), defined as the absence of detectable HCV RNA in the serum 3 months following the completion of therapy [63]. Traditionally, HCV was treated with pegylated-interferon (PEG-IFN) in combination with ribavirin, a nucleoside analogue, demonstrating SVR rates between 30%–60% among chronically infected patients depending on the HCV genotype [64,65]. The introduction of direct-acting antiviral (DAA) therapy in 2011, with the approval of two protease inhibitors, provided promising improvement in SVR rates for patients infected with genotype 1 [66,67,68,69]. The second wave of DAAs, covering more drug classes, resulted in a new IFN-free standard of care for chronic HCV [70]. With current DAA therapies, SVR is achieved in >95% of patients, a considerable improvement from the success rates seen with IFN therapy [1]. These IFN-free regimens offer significantly higher efficacy and tolerability, even in previously challenging populations [71]. Unfortunately, patients continue to clear the virus without sterilizing protection against reinfection [72]. Lack of sterilizing protective immunity means reinfection is common, especially among populations with ongoing risk exposures [73,74,75,76]. The risk of reinfection after effective treatment is reported as 2–6/100 person years among people who inject drugs (PWID) and 10-15/100 person years among human immunodeficiency virus (HIV)-infected men who have sex with men (MSM) [77]. Even these estimates may be low, as they rely on the diagnosis of reinfection, which may include a population more connected to care. Clinical trials have investigated the use of 6 or 8 weeks of DAA regimens during acute HCV infection [78,79,80,81]. Six weeks of treatment with sofosbuvir and ribavirin demonstrated high safety and tolerability, but efficacy was suboptimal with common post-treatment relapse (9/19 participants) [80]. More recent studies investigating the use of ledipasvir and sofosbuvir in acute HCV infection documented high tolerability as well as efficacy [78,79,81]. These trials make a strong case for short-duration treatment of acute HCV infection with potent combination DAA therapy to prevent spread in high-risk populations. However, questions remain about whether timing of treatment and earlier viral clearance could promote protective immunity upon reinfection. 

### 1.4. Evidence of Protective Immunity in HCV Infection

The correlates of protective immunity against HCV infection are not well elucidated. A rapid generation of neutralizing antibodies (nAbs) in the acute phase of infection is associated with spontaneous clearance of infection [82,83], but viral control has also been detected in the absence of high nAb responses [84]. Furthermore, HCV RNA persists in chronic infection with the presence of nAbs [85]. These nAbs are thought to be ineffective as the virus employs effective evasion strategies to escape host immunity. HCV’s RNA Polymerase (NS5B) introduces frequent point mutations generating diversity in nucleotide sequences within quasispecies of a single infected host [86,87]. Glycosylation sites present on envelope glycoproteins (E1 and E2) form a glycan shield to protect functional domains and, consequently, limit access of epitopes to nAbs [86]. Furthermore, interfering Abs and the ability of the virus to disseminate cell-to-cell hinders the action of nAbs [86,88]. Together, these results suggest that naturally-produced antibodies alone do not provide protective immunity. 

Despite a lack of sterilizing immunity, studies in chimpanzees and humans have demonstrated evidence of protective immunity following spontaneous clearance of HCV [24,89,90]. Reinfection of chimpanzees with HCV demonstrates lower peak viremia and more efficient resolution of infection compared to primary infection [24]. Antibody-mediated depletion of memory CD8^+^ T cells before a third infection resulted in an established infection, confirming the role of CD8^+^ T cells in clearance of reinfection [24]. Among people who inject drugs (PWID), the duration and maximum level of viremia during reinfection were decreased, compared with their primary infection [89]. The demonstration that spontaneous clearance without the development of T cell exhaustion leads to levels of protective immunity suggests optimizing the timing of treatment to maximize the reversal of or even prevent T cell exhaustion could be valuable [90]. 

### 1.5. Reversing Immune Dysfunction with Blockade of Exhaustion Markers

There has been considerable interest in avoiding and/or reversing immune exhaustion to encourage improved outcomes for patients with HCV infection. The blockade of PD-1 has been evaluated in vitro and shown to increase the response of peripheral blood-derived HCV-specific CD8^+^ T cells to HCV peptide stimulation [41,46]. However, PD-1 blockade failed to restore the function of HCV-specific CD8^+^ T cells that were isolated from liver biopsies [91]. Subsequent studies demonstrated that the restoration of intrahepatic T cell function required simultaneous blockade of several inhibitory molecules including CTLA-4 and TIM-3 [53,92].

Anti-PD-1 antibodies have been used to block PD-1 signaling in both HCV-infected chimpanzees [93] and in human patients with chronic HCV infection [94]. Fuller et al. showed an increase in HCV-specific CD8^+^ T cell responses and a significant but transient reduction in HCV viremia in one of three chimpanzees [93]. This animal had the strongest and broadest CD4^+^ and CD8^+^ T cell response prior to development of chronic infection, which suggests that PD-1 blockade alone is not sufficient to achieve viral clearance [93]. Gardiner et al. demonstrated partial success of PD-1 blockade in a human population [94]. Five patients who received anti-PD-1 antibodies and one placebo patient demonstrated a reduction in HCV RNA ≥0.5 log10 IU/mL on at least 2 consecutive visits [94]. Three patients who received the PD-1 blockade achieved a >4 log10 reduction. Two patients who received the PD-1 blockade achieved HCV RNA below the lower limit of quantitation, one of which remained RNA-undetectable one year after the study was completed [94]. The observed reductions in HCV replication persisted for more than eight weeks in the majority of patients demonstrating continued reversal of exhaustion [94]. Besides exhaustion marker blockade, researchers have also explored the potential to leverage HCV treatment to restore immune function. 

## 2. Improved Immune Restoration in Acute vs. Chronic IFN-α Treatment

Spontaneous clearance of acute HCV infection is characterized by the presence of HCV-specific CD8^+^ T cells expressing memory marker IL-7Rα (CD127) and anti-apoptotic marker Bcl-2 with strong functional responses measured by cytokine secretion [95]. Spontaneous resolution correlates with early development of IFN-γ- and IL-2-producing and CD107a^+^ virus-specific CD8^+^ T cells [95]. A body of literature suggests that this phenotype can be preserved and/or re-established when IFN-α therapy is administered during acute HCV infection, but not during chronic HCV infection [95,96,97]. Several studies have investigated immune restoration following IFN-α therapy, the previous standard of care for HCV infection. Vertuani et al. found that following IFN-α therapy with or without ribavirin, patients who successfully cleared chronic HCV infection exhibited significantly stronger HCV-specific CD8^+^ responses than the untreated patients with chronic HCV infection [98]. The majority of treated patients showed CD8^+^ responses to at least 3 HCV specific epitopes, demonstrating a CD8^+^ response directed against more epitopes compared to the untreated group [98]. Morishima et al. also demonstrated that HCV-specific cytolytic responses measured by chromium release limiting dilution assay are found more commonly with IFN treatment (with or without ribavirin)-induced control of viremia measured 6 months post-treatment compared to individuals chronically infected with HCV [99]. Tatsumi et al. looked at the frequencies of HCV-specific CD8^+^ T cells following combination therapy of PEG-IFN-α with ribavirin in patients with chronic HCV infection. Their results demonstrated a significant increase of HCV-specific CD8^+^ T cells at 4 weeks after the initiation of treatment compared to frequencies measured in the same patient group pre-treatment [100]. This increase may be associated with elimination of HCV [100]. Furthermore, they suggest that specific reactivity to Core and NS3 protein-derived peptides may predict clearance of the virus with IFN treatment [100]. Kamal et al. focused on HCV-specific CD4^+^ T_h_1 responses in patients chronically infected with HCV [101]. This study found that patients who achieved SVR following PEG-IFN-α therapy with or without ribavirin, maintained multispecific HCV-specific CD4^+^ T-cell responses with enhanced IFN-γ production [101]. In contrast, the HCV-specific CD4^+^ T_h_1 responses in patients who relapsed or only partially responded to therapy waned or were lost [101]. 

Interestingly, Caetano et al. reported that treatment-naïve chronically HCV-infected patients who would eventually achieve a sustained response to IFN therapy showed significantly stronger HCV specific CD8^+^ T cell response than non-responders prior to therapy [102]. In responder patients, terminally differentiated effector cells increased more rapidly, and their frequency was always higher than in non-responder patients. Sustained-responder patients also showed a higher frequency of HCV-specific CD8^+^ T cells producing cytotoxic factors including perforin and granzyme B involved in cell death by lysis and apoptosis.

Badr et al. analyzed a cohort of patients who resolved acute infection following early treatment and documented the phenotype and function of HCV tetramer-specific cells prior to, during and up to 1 year following completion of PEG-IFN-α antiviral therapy with no ribavirin [95]. Early IFN therapy reconstituted a T cell memory response with the same phenotypic and functional characteristics as memory T cells induced following spontaneous clearance [95]. Upregulation of CD127 and Bcl-2 upon viral elimination was documented, and remained detectable 58 weeks post-treatment. PD-1 expression on HCV-specific T cells was downregulated in all patients upon viral clearance [95]. Overall expression of CD127, Bcl-2 and PD-1 was not affected in total CD8^+^ T cells and control staining using cytomegalovirus (CMV) and flu tetramers demonstrating the phenotypic observation was exclusive to HCV-specific T cells [95]. 

Abdel-Hakeem et al. compared reconstitution in early versus late IFN-α therapy without ribavirin [96]. CD8^+^ T cell proliferative responses were higher in patients treated during acute versus chronic HCV infection, and did not differ significantly when acutely treated patients were compared to those who spontaneously cleared HCV [96]. HCV-specific IFN-γ producing responses by CD4^+^ and CD8^+^ T cells were higher in the acute treatment group compared to patients who were treated in chronic HCV infection and achieved SVR [96]. Missale et al. compared HCV-specific CD8^+^ T cell functional restoration as evidenced by IFN-γ analysis directly ex vivo after PEG-IFN-α treatment with or without ribavirin in individuals with acute and chronic HCV infection [97]. Mean frequency of IFN-γ-positive HCV-specific CD8^+^ T cells was higher among acutely treated individuals compared to those treated in chronic HCV infection [97]. In contrast, the frequency of IL-2 positive HCV-specific CD8^+^ T cells was similar in individuals treated during acute HCV infection, individuals treated during chronic HCV infection and spontaneously-resolving groups [97]. IFN-γ production by HCV-specific CD8^+^ T cells after in vitro expansion, proliferative ability and cytotoxic T cell function was stronger in patients with an acute HCV infection compared to those with a chronic HCV infection and more closely resembled self-resolvers [97]. Proliferative capacity was also measured during acute phase of infection and after treatment. This demonstrated restoration of proliferative capacity post treatment in 3 of 4 patients tested [97]. These results are in line with Abdel-Hakeem et al. [96], and suggest an incomplete functional restoration following treatment of acute HCV infection despite improvement with respect to treatment of chronic HCV infection. 

Collectively, these data suggest that IFN-induced SVR leads to partial, but incomplete restoration of HCV-specific immune responses. Restoration is more marked when HCV is treated during the acute phase of infection, possibly because treatment occurs before the development of exhaustion. One confounding factor is the effect of IFN-α itself as it has inhibitory effects on lymphocytes, including both CD4^+^ and CD8^+^ T cells, rendering it difficult to definitively determine if the IFN-α therapy or viral exposure is responsible for changes in phenotype [103,104]. IFN-α blocks S-phase entry of stimulated T lymphocytes inhibiting proliferation [103]. IFN production can be induced endogenously by poly(I · C), a mismatched double-stranded RNA. Virus-induced suppression of proliferation can be replicated by contact with the poly(I · C), while no poly(I · C)-induced impairment in proliferation can be seen in cells lacking IFN-α/β receptors [105]. The introduction of highly effective IFN-free second-generation DAA treatments could help clarify the question of whether IFN-based therapy itself or long-lasting exposure to ongoing viral replication mediates T cell impairment.

## 3. Unclear Outcomes for Immune Restoration following Direct-Acting Antiviral (DAA) Therapy

With the introduction of DAA therapies in 2011, we can now study immune restoration in the absence of IFN. DAA therapy has been shown to restore HCV-specific CD8^+^ T cells in patients with chronic HCV infection [106]. Martin et al.’s cohort of individuals with chronic HCV infection was treated with a combination of faldaprevir and deleobuvir with or without ribavirin [106]. Significant increases in the frequency of HCV-specific CD8^+^ T cells were seen at follow-up compared to baseline after in vitro expansion in the majority of patients that achieved SVR12 [106]. Successful DAA treatment was associated with an increase in CD127 expression on initially CD127-negative HCV-specific CD8^+^ T cells in two patients [106]. Furthermore, a decrease of mean PD-1 expression on HCV-specific CD8^+^ T cells from 33.9% at baseline to 18.0% at follow up was documented [106]. Burchill et al. documented a reconstitution of the CD4^+^ T cell compartment and partial reversal in exhaustion markers on HCV-specific T cells following DAA treatment in patients with chronic HCV infection [107]. This restoration was apparent through an increase in CD4^+^ T cell numbers and a temporal increase in the proliferative response to T cell receptor (TCR) stimulation [107]. A reduction in expression of co-inhibitory molecule, T cell immunoreceptor with immunoglobulin and immunoreceptor tyrosine-based inhibition motif domains (TIGIT) on all T lymphocytes was also documented [107]. A partial reversal in exhaustion phenotype was found in HCV-specific CD8^+^ T cells with a reduction in PD-1 expression, but no increase in frequency of circulating HCV-specific CD8^+^ T cells [107]. The percentage of NK cells expressing TIM-3 was reduced following DAA therapy, and circulating NK cells shifted towards a differentiated, more functionally active population shown through reduction in T-bet and increase in Eomes, two T-box transcription factors [107]. 

A restoration in T-cell activation was documented in a recent study by Emmanuel et al. among HCV-monoinfected and HIV/HCV co-infected patients treated with DAA therapy during chronic HCV infection [108]. The study found increasing recovery in T-cell activation during DAA therapy and post-SVR [108]. Before DAA therapy, HCV-monoinfected patients had higher CD4^+^ and CD4^+^:CD8^+^ T-cell ratio [108]. An observed decrease in activated CD4^+^ and CD8^+^ T cells in both monoinfected and coinfected patients from pretreatment to post-SVR suggests clearance of HCV normalizes activated T-cell levels [108].

Wieland et al. investigated the differentiation and outcome of HCV-specific CD8^+^ T cells after DAA therapy [109]. HCV-specific CD8^+^ T cell populations from patients chronically infected with HCV displayed a heterogeneous phenotype that included CD127^+^ PD1^+^, CD127-PD1^lo^ and CD127-PD1^hi^ subsets [109]. This study also examined the expression of T cell factor 1 (TCF1), which is a transcription factor required for the differentiation and persistence of memory CD8^+^ T cells. A larger proportion of CD127^+^PD1^+^ HCV-specific T cells expressed TCF1 compared to the other subsets. These TCF1^+^CD127^+^PD1^+^ T cells were maintained during and after DAA-induced HCV elimination and showed memory-like characteristic including survival in the absence of HCV antigen and recall proliferation after HCV viral relapse. Peptide-expanded HCV-specific CD8^+^ T cells derived from patients at the completion of DAA therapy exhibited increased IFN-γ production compared to baseline suggesting some immune restoration in patients following DAA treatment of chronic HCV infection [109]. 

However, in a study by Zhang et al. the authors found no functional immune reconstitution of HCV-specific T cells after DAA treatment in patients chronically infected with HCV [110]. Although DAA treatment of chronic HCV infection led to SVR in all patients, immune reconstitution was not documented [110]. This cohort was composed of both HCV-1b and HCV-2a chronically infected patients who were given 12 or 24 weeks of combination DAA therapies [110]. Successful treatment did not improve antigen-specific CD8^+^ T cell IFN-γ production with all patient groups changing between time points presenting no apparent trend [110]. Phenotyping experiments did not reveal any significant differences in PD-1 expression before and after treatment for both CD4^+^ and CD8^+^ T cells [110]. The reasons for these differences in results compared to the earlier DAA studies in patients chronically infected with HCV is not clear and suggests that larger studies may be warranted. 

Some indirect evidence of immune recovery after HCV clearance was demonstrated in a cohort of patients with chronic HCV-induced cryoglobulinemia vasculitis who were treated with DAA therapy. Successful HCV clearance with DAA therapy has been shown to reverse disturbances in peripheral B- and T-cell populations [111]. In HCV-associated cryoglobulinemia vasculitis, Follicular Helper T cells (T_FH_) expansion has been associated with T_h_1 and T Helper 17 Cells (T_h_17) polarization, and expansion of IgM^+^CD21^−/low^ memory B cells and low levels of T_reg_ cells [111]. DAA therapy improves irregularities in B-cell homeostasis, with a decreased percentage of autoreactive memory B cells and cryoglobulin levels post therapy [111]. Furthermore, successful anti-HCV therapy restores T-cell homeostasis by reestablishing T_h_1/T_h_17 balance and improving T cell activation [111]. 

During an infection with HCV there is a marked increase in intrahepatic and peripheral IFN-stimulating genes (ISGs) [112,113,114]. The majority of ISGs have antiviral properties, but upregulation during a chronic infection with HCV is ineffective at clearing the virus [112,113,114]. The inability of the host to clear HCV with increased ISG expression suggest an exhausted immune phenotype characterized by a refractory state of IFN-signaling [112]. Holmes et al. investigated changes in innate immune response during and after DAA therapy administered during chronic HCV infection [115]. ISG expression was found to be downregulated at week 2 and 4 of DAA treatment, but recovered by the end of treatment to a level below that observed at baseline [115]. ISGs quantified at SVR showed levels similar to treatment week 2 [115]. This second downregulation of ISG following DAA treatment, suggests a reversal of the exhausted ISG phenotype [115]. NK cell activity, a key component of the innate immune response, is reduced in individuals chronically infected with HCV [116]. Corado et al. documented that spontaneous NK cytotoxicity was four-fold lower in patients chronically infected with HCV than in healthy donor demonstrating a significant functional impairment [116]. There are two major populations of NK cells, CD56^dim^NK and CD56^bright^NK [117,118,119]. CD56^dim^ NK cells are more cytotoxic than CD56^bright^NK cells [120], but produce significantly less IFN-γ [121]. It was reported that a CD56^dim^NK cell subset, but not a CD56^bright^NK cell subset, showed significantly lower frequencies in patients with chronic HCV compared to healthy subjects [122]. DAA therapy in chronic HCV patients has been shown to improve NK activity by increasing the frequency of CD56^dim^NK cells at SVR24 [123]. It was also found that NK activity quantified by Chromium-50 release assay significantly improved at end of treatment versus prior to therapy (*p* < 0.01) and at follow up 24 weeks post treatment (SVR24) versus prior to treatment (*p* < 0.001) in 30 patients [123]. 

## 4. Conclusions and Future Perspectives

**Figure 1:**
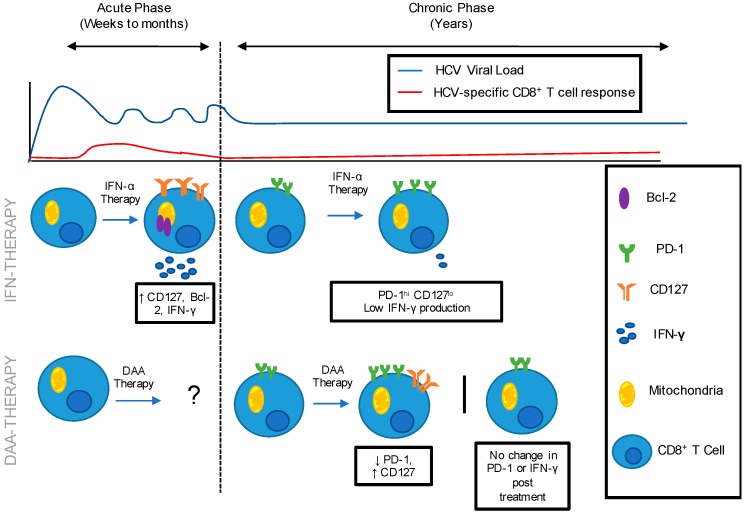
HCV-specific Immune Restoration following IFN and DAA therapy administered during the acute and chronic phase of HCV infection. HCV is characterized by an acute phase of infection in which the immune response is unable to control HCV replication, leading to chronicity in the majority of patients. Following IFN-α therapy administered during acute HCV infection: CD8^+^ T cell populations expressed low levels of exhaustion marker, PD-1 and increased levels of memory marker CD127, anti-apoptotic marker Bcl-2 and IFN-γ production. Following IFN-α therapy administered during chronic HCV infection: CD8+ T cell populations expressed high level PD-1 and low level of CD127 and IFN-γ production. Immune restoration following DAA therapy administered during acute HCV infection has not been documented in the literature. Following DAA therapy administered during chronic HCV infection: Literature demonstrates varying results. Decreased PD-1 and increased CD127 has been reported, while no change in PD-1 expression post-treatment has also been reported.

IFN-based treatment during acute HCV infection has demonstrated an improved likelihood of immune restoration compared to patients treated in chronic HCV infection. Figure 1 shows an inverse relationship between the level of immune restoration and the duration of exposure to HCV antigen. DAA treatment has led to some level of immune restoration when administered to patients chronically infected with HCV, but results are variable and require further investigation (Figure 1). Reversal of HCV-specific immune exhaustion results in treated patients acquiring an immunological phenotype similar to spontaneous clearers. Combining these results, there is reason to believe that DAA treatments will outperform IFN therapy in their capacity to rescue exhausted T cells upon HCV clearance during acute infection. 

Complicated molecular pathways determine the differentiation of CD8^+^ T cells into memory or exhausted T cells during HCV infection [26]. In a prospective study, the transcriptional profiles of HCV-specific CD8^+^ T cells from HCV infected patients progressing to persistent infection and patients spontaneously resolving HCV infection were compared during the acute phase of infection [26]. Dysregulation of metabolic processes during acute infection was observed in patients who progressed to persistent infection [26]. This dysregulation was linked to changes in gene expression related to cellular pathways including nucleosomal regulation of transcription, T cell differentiation, and the inflammatory response [26]. These changes in HCV-specific CD8^+^ T cell transcription came before the establishment of T cell exhaustion, suggesting it as a target the origins of T cell exhaustion in chronic HCV infection [26]. Further research and understanding in this field, may help direct treatment to improve restoration and lower dysfunction. 

It is difficult to determine if this reconstitution is the cause or effect of enhanced viral clearance. It is hypothesized that it is the consequence of clearance, as T cells are being further exhausted by continued antigen exposure. Large studies to track reinfection following DAA treatment at different stages of infection are necessary to see the outcome of protective immunity. The studies included in this review typically utilize peripheral immune cells and it is not fully understood how the function of peripheral and liver-infiltrating immune cells parallels each other. Recent investigation suggests HCV-specific T cell responses in the periphery do not reflect those in the liver [124]. 

Presently, DAA treatment is generally initiated during chronic HCV infection to avoid treating patients who may spontaneously clear the infection without treatment. Based on current research, we identify the need to investigate immune restoration following early treatment of HCV infection with current DAAs. We hypothesize that DAAs administered during acute infection may prevent progressive T cell exhaustion characteristic of chronic HCV infection, leading to enhanced T cell memory and improved protection against progression to chronicity upon subsequent HCV reinfection. Ongoing clinical trials testing the efficacy of DAA treatment for recently-acquired HCV infection, including the REACT Trial (Randomized Study of Interferon-free Treatment for Recently Acquired Hepatitis C in People Who Inject Drugs and People with HIV Coinfection, (https://clinicaltrials.gov/ct2/show/NCT02625909) may be leveraged to explore immune restoration following DAA therapy administered in early HCV infections. The REACT trial includes a long follow-up period to monitor cases of reinfection among this population. Data and samples generated from this trial may be used to study HCV-specific immune restoration following therapy during acute HCV infection and investigate evidence of protection against progression to chronicity after reinfection.

The potential for immune restoration with early treatment may have additional clinical benefits, including a reduced risk of hepatocellular carcinoma (HCC) development. The risk of HCC after DAA-induced SVR is unclear. A recent study found that among patients treated with DAA, SVR was associated with a reduction in the risk of HCC compared to patients who did not achieve SVR [125]. As the majority of infected individuals do not develop specific symptoms, improved screening methods to identify unknowingly infected individuals is necessary. Future findings could provide the foundation for informing treatment guidelines for early HCV infection and improve our understanding of HCV-induced T cell exhaustion while further elucidating the factors associated with protective immunity among these patients. While studies continue to address the barriers related to the development of an HCV vaccine, early treatment may be used to rescue immune responses and reduce reinfection rates.
